# The British Medical Almanack, and Supplement

**Published:** 1836-01

**Authors:** 


Art. XIII.—*The British Medical Almanack, and Supplement.* 1836. *London.* Small 8vo. Pp. 160.

The industry of the editor, and the exertions of the publisher of this excellent annual, are equally conspicuous in this, which is the second year of the appearance of this almanack. It is literally *full* of information, admirably selected and arranged. To the calendar is appended a chronological history of medicine. Lists are given of all the public medical bodies, and of the medical officers of nearly every public institution in the kingdom. Numerous useful particulars are inserted relating to all the universities and schools of medicine, and to various learned societies. The lecturers, the fees, the hours of lecture, of all the great schools of London, are stated in tables; and the expence of attending the hospitals, of dresserships, &c., are given in a similar manner. Accurate information has been collected for its pages, concerning all the provincial schools, and many provincial societies; as well as concerning the Faculty of Medicine of Paris. Indeed there is scarcely a subject on which occasional information is required, either by practitioners or by students, in relation to the general state of the profession, which is not here to be found.

In the Supplement, now for the first time added, we observe numerous notices of statistical interest; an account of English and French weights, and of new medicines; of tests of the urine, and the hydrometer; a short but correct and useful description of the signs derived by auscultation and percussion, with a tabular view of them; a list of antidotes of several of the poisons, and directions for the treatment of asphyxia; with an abstract of the Anatomy Act. The statistical tables relate to the mean weight and height of the human body at all ages; the weight of the skeleton, brain, and heart; the laws of human mortality; the average number of *females* dying annually; the mortality and diseases of armies; the proportion of sickness at different ages; statistics of the English hospitals, (*this seems particularly deserving of the attention of the medical officers of town and country hospitals*); statistics of the epidemic cholera in England (the *Facts* by the late Sir David Barry); the bills of mortality; tables illustrating the laws of disease, &c. &c. On many of these subjects, it is well known to practitioners, particularly in the country, that correct information is not readily to be attained: that given in the supplement to the British Medical Almanack bears every mark of having been prepared with the utmost care. We are justified in saying that there is no existing work in which so many important particulars are condensed into so very cheap a book. The work is got up with great neatness, and independent of its *professional* merits, answers the purpose of a very convenient calendar for the meteorologist and botanist. A medical almanack has long been wanted, and a more useful one than this could hardly be imagined.

